# What Australian young adults with disability are prepared and willing to pay to exercise in a gym: An online survey and discrete choice experiment

**DOI:** 10.1016/j.jsampl.2026.100154

**Published:** 2026-09-10

**Authors:** Rachel A. Kennedy, Utsana Tonmukayakul, Nora Shields

**Affiliations:** a Olga Tennison Autism Research Centre, La Trobe University, Bundoora, Victoria, 3086, Australia; b Deakin Health Economics, School of Health and Social Development, Institute for Health Transformation, Deakin University, Geelong, 3220, Australia

**Keywords:** Community gyms, Fees, Access, Inclusion, Physical activity

## Abstract

An online survey and discrete choice experiment determined prepared budget and willingness-to-pay for gym access among young adults with disability. Young adults were prepared to pay $20/week (median) to access a gym. Mixed logit model fit statistics determined willingness-to-pay for four entry options. Bootstrapped willingness-to-pay and contemporary (‘market reality’) gym fee data provided estimates for these options: $9/session casual entry to gym, $14.23/session casual entry to gym plus other facilities, $18.65/week for unlimited gym entry (membership) and $23.57/week for unlimited entry to gym plus other facilities. Findings suggested “value-added” memberships are likely unaffordable for young adults with disability.

## 1 Introduction

Gyms are popular for exercise and socialisation among young adults [1,2]. A recent study of contemporary community gym practices including entry fees and options, identified an industry emphasis on “value-added” all-included memberships while disincentivising casual one-off gym entry [3]. Young adults with disability are willing to pay for gym access but prefer the flexibility of casual entry [3]. However, current gym practices create barriers for young adults with disability seeking recreational and social engagement [2,3]. To our knowledge, no published economic analysis has examined what young adults with disability are prepared and willing-to-pay to access a gym.

A prepared budget reveals how people plan to allocate their money based on what they value most. Willingness-to-pay captures the maximum value individuals place on goods or services, measuring preferences in monetary terms. Measuring prepared budget and willingness-to-pay identifies the gap between what young adults with disability value, what they plan to spend, and actual gym entry fees (‘market reality’), which are important for developing equitable gym pricing.

Among young adults with disability, our objectives were to.

1) Determine their prepared budget for gym entry; and
2) Estimate their willingness-to-pay for four gym entry options and price estimates for each option:
  • one-off (casual) gym entry only
  • one-off (casual) gym entry plus additional facilities (for example, pool access and/or fitness classes)
  • weekly unlimited gym entry (membership), and
  • weekly unlimited gym entry (membership) plus additional facilities.

## 2 Methods

An anonymous online survey and discrete choice experiment was conducted between May and August 2023 (Appendix A). Ethics approval was obtained prior to study commencement. A convenience sample of Australian young adults with any disability aged 18–35 years were recruited through social media, flyers, and emails distributed by disability, sport and recreation organisations. Consent was implied through survey completion. The survey asked about demographics, gym attendance, and the highest price respondents were prepared to pay for gym entry (per casual visit and per week for unlimited membership). A discrete choice experiment assessed respondents’ preferences for different gym entry options [4,5]. We used QuestionPro’s discrete choice model to generate six choice tasks in randomised order using a full factorial experimental design, allowing for estimation of main effects and all possible interactions between attributes. A sample question preceded the six choice tasks to show respondents how the choice task worked; the practice question was subsequently removed from the analysis. It is recognised that recruiting people with disability for qualitative research can be difficult [6]. Therefore, given the anticipated modest sample size, the analysis estimated main effects only to ensure model parsimony and reliable parameter estimates. Entry fee was modelled with a linear functional form. No blocking or status quo option were used. Each choice task included two hypothetical gym options with different attribute combinations. Each choice task (see Appendix A for an example), presented three attribute combinations derived from a previous study [3] and face-validation with a consumer group of six young adults with disability. Face-validation included piloting, subjective understanding, respondent burden, timing and wording. Based on the feedback of the consumer group, no changes were made to the discrete choice experiment structure, which was as follows.

1. Entry type with 2 levels (casual or membership)
2. Facilities with 2 levels (gym only, or gym plus additional facilities)
3. Entry fee with 3 levels conditional on entry type (casual entry: $10, $18, $26 per session; membership: $17, $21, $24 per week for unlimited access). Price levels were derived from reported contemporary gym fees [3].

### 2.1 Analysis

Descriptive statistics characterised the survey respondents. Data were tested for normality (Shapiro–Wilk test) and statistical tests applied accordingly. Market reality casual entry fees of $6-$13 were estimated from an earlier study [3] and reflected national benchmarks (median $5 and mean $11 per entry) [1]. Casual entry fees were converted to weekly costs by multiplying by 1.87, the weighted average number of times Australian adults with disability participated in sports activities per week [1,2]. This conversion allowed direct comparison between casual entry costs, weekly memberships and respondents weekly prepared budget. Using reported participation frequency data ensured the cost comparison reflected authentic usage patterns.

To enable robust statistical comparisons between market reality entry fees and respondents’ prepared budget, bootstrapping, a random sampling with replacement method using the original observed data [7], was performed using Microsoft Excel and STATA 18 statistical software to generate 1000 random observations from the observed datasets.

Willingness-to-pay for gym entry options were determined using a discrete choice experiment. Entry type and facilities attributes were coded as binary variables whereas entry fee was treated as a continuous variable. Choice data were analysed using a mixed logit (MXL) model with the *logitr* package in R [8]. The MXL specification modelled entry type (casual vs membership), facilities (gym only vs gym plus additional facilities) as random parameters with normal distributions to capture preference heterogeneity across respondents, while the entry fee attribute was treated as a fixed parameter. This specification allows for individual variation in preferences for gym features while maintaining a common price sensitivity, consistent with standard practice in discrete choice experiments [9]. The MXL model demonstrated superior fit compared to a multinomial logit (MNL) model base on log-likelihood (see Footnote^1^). Willingness-to-pay estimates for each gym entry option were calculated using the marginal rate of substitution method, dividing the non-monetary attribute coefficients by the price coefficient, with casual gym-only entry as the reference category. Given the modest sample size, the willingness-to pay estimates were bootstrapped using 1000 iterations to provide more robust and reliable confidence intervals for the premium amounts respondents were willing to pay above the reference casual entry fee.

## 3 Results

Fifty-four young adults with disability (mean age 25.9 years (SD 5.5), range 18–35 years) responded (Table 1). Of note, 23 respondents (43%) lived in a postcode with the highest (10th) Index of Relative Socio-economic advantage and disadvantage (IRSAD) decile while eight (15%) lived in a postcode with the lowest (1st) decile. Thirty-one (57%) were in paid employment; 19 (41%) reported a fortnightly after-tax income below the poverty line (<$1000/fortnight) [10]. Thirty-three attended a gym, 44 provided data to calculate prepared budget and 53 completed the discrete choice experiment to estimate willingness-to-pay. Thirty current gym attendees paid casual gym entry ranging from $5 to $50 (the latter for personal training; mean $15.62 (SD $10.25)). All available data were included in the analysis.

**Table 1:** Characteristics and demographics of survey respondents.

Characteristic	N = 54 (%)
**Sex**	
Female	29 (54%)
Male	20 (37%)
Non-binary or self-described	5 (9%)
**Type of disability***(note, respondents could provide more than one answer)*	
Physical	28 (52%)
Intellectual	22 (41%)
Psycho-social	16 (30%)
Sensory	10 (19%)
Autism or ADHD	5 (9%)
Neurological (other)	2 (4%)
**State in which they reside**	
Victoria	50 (92%)
New South Wales	2 (4%)
South Australia	2 (4%)
**Location**	
Metropolitan	44 (81%)
Regional	8 (15%)
Rural	2 (4%)
**IRSAD**	
5th decile (relative advantage)	44 (81%)
≤5th decile (relative disadvantage)	10 (19%)
**Residing with**	
Family	36 (67%)
Partner or spouse	8 (15%)
Alone	6 (11%)
Share house with others	3 (6%)
Supported accommodation or independent living with a carer	1 (2%)
**Highest level of education achieved**	
Primary school	1 (2%)
Some high school, did not complete	8 (15%)
High school certificate	16 (30%)
Vocational training/TAFE	8 (15%)
Higher education/university	18 (33%)
None of the above	3 (6%)
**Employment**	
Paid (breakdown of type of employment in italics)	31 (57%)
*Part-time*	*15*
*Casual*	*10*
*Full-time*	*6*
Not in paid employment	23 (43%)
**Income support***(note, respondents could provide more than one answer)*	
Disability support pension	27 (50%)
Other	36 (67%)
No income support	16 (30%)
**Concession card holder***(note, respondents could provide more than one answer)*	
Pension card	21 (39%)
Other type of concession card	42 (78%)
No concession card	16 (30%)
**Fortnightly after-tax income**	
$1-250	5 (9%)
$251-500	3 (6%)
$501-750	6 (11%)
$751-1000	5 (9%)
$1001-1250	14 (26%)
$1251-1500	5 (9%)
$1501-1750	1 (2%)
$1751-2000	3 (6%)
More than $2000	4 (7%)
Prefer not to say	8 (15%)
**NDIS participant***(52 provided a response)*	43 (83%)

IRSAD Index of Relative Socio-economic advantage and disadvantage, TAFE Technical and Further Education, NDIS National Disability Insurance Scheme.

Survey respondents reported paying higher weekly fees (median $28.05 per week, IQR 9.35 range $9.35- $93.50), likely reflecting the sample’s relatively high socioeconomic bias [11]. Therefore, the reported $6-$13 range [3] was adopted for subsequent analyses as it represents contemporary market reality pricing.

The median prepared budget of young adults with disability was $20 per week (IQR 10, range $5-$50), with a significantly skewed distribution (*p* < 0.001).

The MXL yielded coefficients of 9.23 (95%CI 6.58, 11.90, *p* < 0.001) for membership entry and 4.94 (95%CI 2.12, 7.73, *p* < 0.001) for additional facilities, with a fixed price coefficient of −1.00. As willingness-to-pay equals the coefficient divided by the absolute price coefficient, respondents were willing to pay an extra $9.23 per week for membership and $4.94 for additional facilities. These willingness-to-pay premiums were added to the market price benchmarks of $6 - $13^3^ (base price) to estimate four entry options, for instance gym membership is base market price plus $9.23 premium. Therefore, the median casual gym entry became $9, casual gym entry with additional facilities access $14.23, weekly membership $18.65, and weekly membership with additional facilities $23.57 (Table 2).

**Table 2:** Estimated median fees (interquartile range, range) for four gym entry options as calculated with bootstrapping from willingness-to-pay and market reality data.

Types of gym entry	Median	IQR	Range	Shapiro Wilk normality test (*p*-value)
Casual gym entry only (reference) per session	$9	$7 -$11	$6 - $13	<0.001
Casual gym entry with additional facilities^a^ per session	$14.23	$12.23 - $16.41	$7.17 - $20.85	<0.001
Weekly unlimited gym membership	$18.65	$16.54- $20.59	$11.52 - $25.56	<0.001
Weekly unlimited gym membership with additional facilities^a^	$23.57	$21.40 - $25.67	$14.82 - $31.95	<0.0059

a Additional facilities may include access to pool, fitness classes and other recreation facilities.

## 4 Discussion

“Value-added” memberships favoured by the gym industry are likely unaffordable for many young adults with disability. While 61% of respondents currently attend gyms, direct comparison of our findings reveals critical affordability gaps. The median prepared budget was $20 per week, yet estimated fees for membership with additional facilities ($23.57) exceeded this limit. More concerning, 41% of respondents earned below the poverty line [8], meaning a $20 weekly gym budget represents 8–10% of their fortnightly income; a substantial proportion for individuals facing multiple competing financial priorities.

Current gym attendance does not contradict affordability concerns but rather highlights the compromises young adults with disability must make. Of the 33 current gym attendees, most paid casual gym entry (mean $15.62/session) rather than membership, suggesting they prioritise flexibility over industry preference for unlimited entry. Casual gym entry alone ($9) fits within budget constraints, but restricts access to additional facilities (pool, fitness classes). Young adults with disability are willing to pay for unlimited membership and additional facilities [3], demonstrating clear demand. However, these additional premiums push total costs beyond financial reach for those earning below the poverty line. Similar to other studies [12,13], survey respondents were unemployed, under-employed or in low-paying jobs, further limiting discretionary spending. Gym entry fees and memberships are not funded by Australian disability schemes [3,14], nor are there universal government measures to support recreation access for young adults with disability. This creates a participation gap where financial barriers force young adults with disability to choose between sporadic casual attendance or forgoing comprehensive gym access.

A strength of this study was the robust analysis combining discrete choice experiment, bootstrapping methods, and market reality pricing data. However, the sample size was small, and respondents were largely from affluent communities, suggesting our findings may underestimate affordability barriers for young Australians adults with disability. A further limitation was the lack of a control group of non-disabled young adults. While understanding gym affordability for non-disabled young adults was beyond the scope of this study, AusPlay data suggests young adults aged 18–34 years paid $9.62 (median) or $13.88 (mean) per week in a similar period (2023–24 fiscal year) [15].

## 5 Conclusion

Young adults with disability are prepared to pay for gym membership and comprehensive facilities, yet financial constraints create barriers. Bridging this affordability gap requires policy intervention such as subsidised access, tiered pricing, or flexible payment structures that match the economic reality of young adults with disability seeking equitable community participation.

## Ethics declaration

Written informed consent to take part in the study and to publish the article has been obtained from all participants or their legal representatives. The privacy rights of participants have been observed.

This study was performed in compliance with relevant laws, regulatory frameworks and guidelines where the research took place. This study was approved by the La Trobe University and Deakin University. (Approval No. La Trobe University Human Ethics Committee (21376) and Deakin University (2022–322))

## Funding information

This study was supported by a Victorian Health Promotion Foundation (VicHealth) Impact Research Grants Initiative (2021).

## Declaration of competing interest

The authors have no conflict of interests to declare.

## Footnotes

1 Model fit statistics: mixed logit model (MXL) showed superior fit with log-likelihood −107.76 vs multinomial model (MNL) −129.02: McFadden’s R^2^ 0.45 vs 0.34 (adjusted 0.42 vs 0.32): Akaike information criterion 225.53 vs 264.04; Bayesian information criterion 243.74 vs 274.96.
