# Supplementary material for: What Australian young adults with disability are prepared and willing to pay to exercise in a gym: An online survey and discrete choice experiment
Source: JSAMS Plus. 2026 Sep 10;8:100154. doi: 10.1016/j.jsampl.2026.100154 (PMC13586742; doi:10.1016/j.jsampl.2026.100154)
Supplement: Supplementary file 1 [file mmc1.pdf]

Discrete choice experiment and survey to determine 'value' of gym access among young people with disability
Brief information statement

1. What is the study about?

We are doing a survey and discrete choice experiment with young people with disability to evaluate their ability and willingness to pay to exercise in a community gym. The discrete choice experiment will establish preferences for gym access under different scenarios and price combinations. For the purposes of this study, a community gym is defined as a publicly funded and owned gym facility that is operated by either the local municipal council or management company or organisation (e.g. Belgravia or the YMCA).

2. Do I have to take part?

Being part of this study is voluntary.

3. Who is being asked to participate?

You have been asked to participate because you are a young person aged 18-35 years who has a disability.

4. What will I be asked to do?

You will be asked to complete an online survey that will take about 10-20 minutes to complete. Your information is kept confidential and will only be available to the research team.

5. What are the benefits?

There are no immediate benefits to you in completing this survey.

6. What are the risks?

We do not believe there are any risks to your involvement.

7. What will happen to information about me?

The information collected from you will include basic information about you (your age and gender, where you live) and information about your disability. This information will always be stored confidentially and handled in accordance with applicable privacy laws and Health Records Act 2001 (Vic). No identifying information will be collected via the survey (such as name). Collected information will be anonymous. There will be no need to de-identify the data.

8. Will I hear about the results of the study?

The results will be published and publicized through the GYM-SPARC project. You can request a copy of the results by contacting the project manager, Dr Rachel Kennedy, phone (03) 9479 2595 or email r.kennedy3@latrobe.edu.au

9. What if I change my mind?

If you no longer want to take part in the survey and discrete choice experiment, or would like to withdraw from the study, you can do so any time prior to completing the survey. All completed surveys are anonymous and we will be unable to identify your survey answers once you have submitted the survey. Your decision to withdraw at any point will not affect your relationship with La Trobe University or any of the research partner organisations.

10. Who can I contact for questions or if I want more information?

If you would like to speak to us, please use the contact details below:

Dr Rachel Kennedy Project manager/Associate investigator phone (03) 9479 2595 or email r.kennedy3@latrobe.edu.au

11. What if I have a complaint?
If you have a complaint about any part of this study, please contact:
Ethics no. HEC21376
Senior Research Ethics Officer (03) 9479 1443 humanethics@latrobe.edu.au

Please contact Dr Rachel Kennedy if you would like a copy of the full information statement.

By proceeding with the online survey, you imply consent for the information you provide to be collected and stored by the research team in accordance with this participant information statement.

Please start the survey now by clicking on the Start button below.

Are you aged between 18 and 35 years and identify as having a disability?

- ◯ Yes
- ◯ No

We wish to understand young people's ability and willingness to pay a gym entry fee. We will use a method called a Discrete Choice Experiment to understand how young people with disability make choice. This involves showing you 2 random options and asking you to select the option you prefer.

The option you choose help us understand the importance of different features of gym entry to young adults with disability when making decisions about how much they are willing to pay.

There are three features of gym entry we are interested in. These are:
1) Casual entry (pay as you go) OR membership (sign up for a membership and you can attend as often as you want)

2) Access to facilities e.g. gym only OR gym & pool & fitness classes
3) The price of the gym entry itself

Suppose that you are deciding to go to a community gym to exercise.
Two options of gym entry, available facilities, and entry fee are shown below.
Each option has different features.
Choose the one you would prefer.

You will be asked to do this **7** times.

Let's get started.

Choose the option you would prefer.

Please note the options are randomly generated. Some of the options might appear to not make sense. We are not trying to trick you.

Remember, you will be asked to do this **7** times.

Step 1 of 7

**Type of gym entry**	**Casual entry (pay per visit)**	**Membership (attend as often as you want)**
**Facilities available**	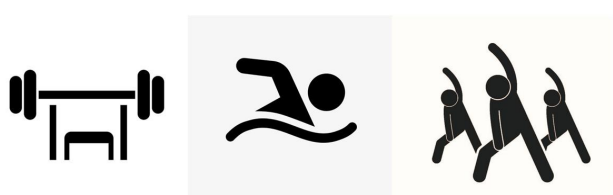 **Gym plus pool and fitness classes**	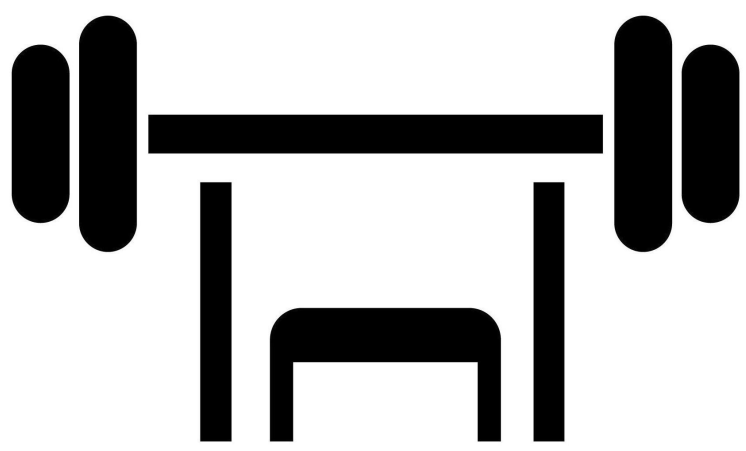 **Gym only**
**Price of gym entry**	**$18 per <u>visit</u>**	**$24 per <u>week</u>**
	○	○

Thank you for answering the Discrete Choice Experiment.

We would now like to know about you.

Please answer the following questions.

* How old are you? (Your age in years)

* What is your gender?

- ○ Male
- ○ Female
- ○ Non-binary
- ○ I prefer not to answer
- ○ Prefer to self-describe (specify)

* Where do you live?

- ○ Metropolitan
- ○ Regional city or town
- ○ Rural
- ○ Remote

* What is the postcode of where you live? (This will not be used to identify you)

* Who do you live with?

- ◯ I live alone
- ◯ I live with my partner or spouse
- ◯ I live with my family
- ◯ I live in supported accommodation/independent living with a carer
- ◯ I live in supported group accommodation/independent living with other people with disabilities and carers
- ◯ I live in a share house with others (Not a supported independent living situation)
- ◯ Other

---

* What is the highest level of school that you have completed?

- ◯ Primary school
- ◯ Some high school, but did not complete Year 12
- ◯ High school certificate
- ◯ Vocational training / TAFE
- ◯ Higher education / university
- ◯ None of the above
- ◯ Other

---

* Are you currently in paid employment?

- ◯ Yes
- ◯ No

---

* If you are in paid employment, which category best describes your workload

- ◯ Full time work
- ◯ Part time work
- ◯ Casual work
- ◯ I am not in paid employment

---

* What income support or benefit/s have you received in the last 3 months? [tick all that apply]

- ☐ Disability support pension

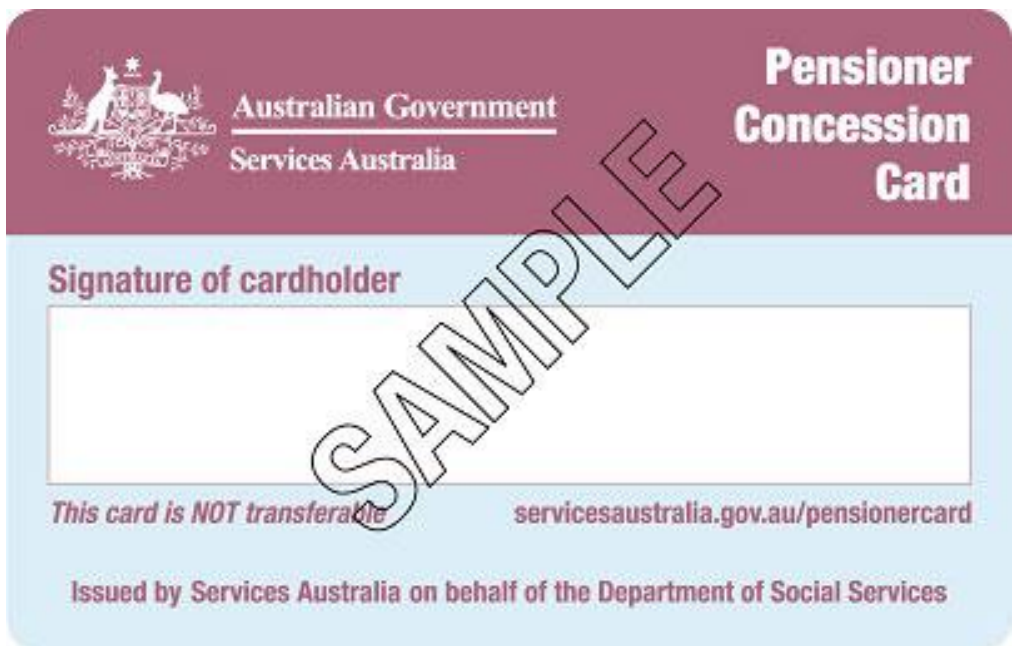

- ☐ Youth allowance
- ☐ Youth disability supplement
- ☐ Sickness allowance
- ☐ Mobility allowance
- ☐ ABSTUDY (for students of Aboriginal or Torres Strait Islander descent)
- ☐ AUSTUDY
- ☐ Unemployment ('New Start' or 'Jobseeker') allowance
- ☐ I do not receive income support
- ☐ Other

---

* What is your fortnightly income after tax (including any paid work and / or pension or allowance)?

- ◯ $1-$250
- ◯ $251-$500

- ◯ $501-$750
- ◯ $751-$1,000
- ◯ $1,001-$1,250
- ◯ $1,251-$1,500
- ◯ $1,501-$1,750
- ◯ $1,751-$2,000
- ◯ more than $2000
- ◯ I would prefer not to say

---

* Do you have a concession card or receive a concession discount?

- ◯ Yes
- ◯ No

---

* If you have a concession card, what type do you have?

- ☐ Healthcare card

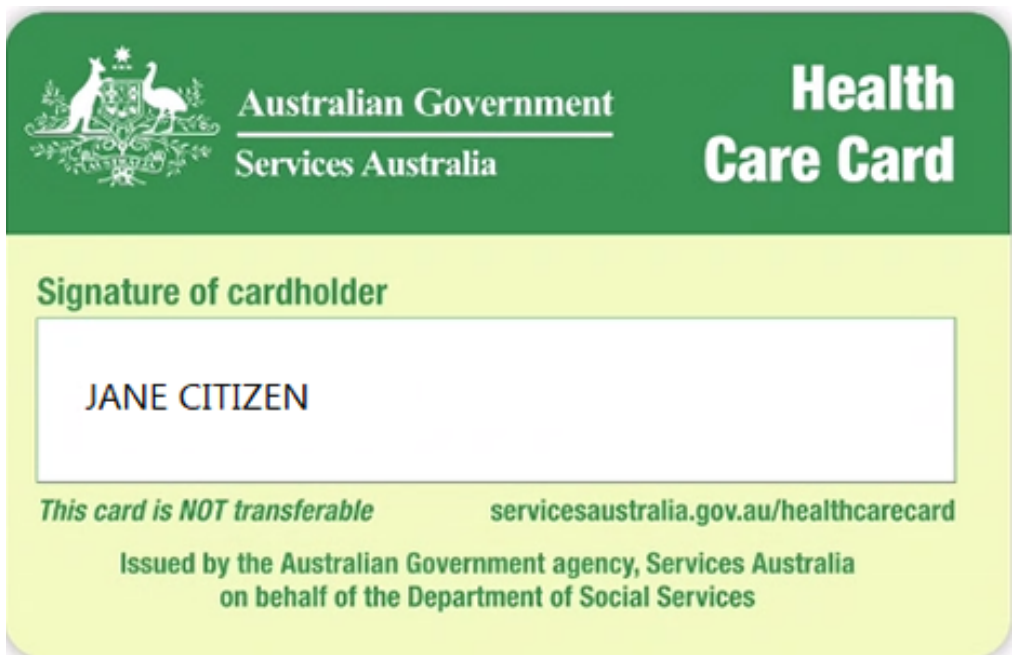

- ☐ Student concession
- ☐ Pensioner concession
- ☐ I do not have a concession card
- ☐ Other type of concession card

* What type of disability do you identify as having? (Tick all that apply)

- [ ] Physical disability, for example: restriction in physical activities or in doing physical work, (difficulty walking or needing to use a wheelchair, difficulty holding or carrying objects, shortness of breath or breathing difficulties that restrict everyday activities, chronic pain, seizures or blackouts)
- [ ] Intellectual disability, for example: difficulty learning or understanding things
- [ ] Sensory disability, for example: loss of sight (not corrected by glasses or contact lenses), loss of hearing where communication is restricted or an aid is used, or speech difficulties
- [ ] Psychosocial disability, for example, nervous or emotional conditions, mental illness, or social and behavioural difficulties that restrict everyday activities
- [ ] Other

---

* Are you a participant in the National Disability Insurance Scheme (NDIS)?

○ Yes

○ No

---

* Do you go to the gym to exercise?

○ Yes

○ No

---

* What is the highest price for gym entry would you be prepared to pay per casual visit?

* What is the highest price for gym membership would you be prepared to pay per week?

Thank you for completing this survey.

If you would like to receive a summary of the findings from this study, please contact the study coordinator.

Dr Rachel Kennedy, GYM-SPARC project manager
Email: r.kennedy3@latrobe.edu.au
Phone: (03) 9479 2595

La Trobe University
